# Identification of novel drug targets for *Helicobacter pylori*: structure-based virtual screening of potential inhibitors against DAH7PS protein involved in the shikimate pathway

**DOI:** 10.3389/fbinf.2024.1482338

**Published:** 2024-10-18

**Authors:** Narjes Noori Goodarzi, Mahshid Khazani Asforooshani, Behzad Shahbazi, Nayereh Rezaie Rahimi, Farzad Badmasti

**Affiliations:** ^1^ Department of Pathobiology, School of Public Health, Tehran University of Medical Sciences, Tehran, Iran; ^2^ Department of Bacteriology, Pasteur Institute of Iran, Tehran, Iran; ^3^ Department of Microbiology, Faculty of Biological Sciences, Alzahra University, Tehran, Iran; ^4^ School of Pharmacy, Semnan University of Medical Sciences, Semnan, Iran; ^5^ Department of environmental Health Engineering, School of Public Health, Shiraz University of Medical Sciences, Shiraz, Iran; ^6^ Student Research Committee, Shiraz University of Medical Sciences, Shiraz, Iran

**Keywords:** *Helicobacter pylori*, structure-based virtual screening, shikimate pathway, DAH7PS, StreptomeDB

## Abstract

**Background:**

*Helicobacter pylori*, a bacterium associated with severe gastrointestinal diseases and malignancies, poses a significant challenge because of its increasing antibiotic resistance rates. This study aimed to identify potential drug targets and inhibitors against *H. pylori* using a structure-based virtual screening (SBVS) approach.

**Methods:**

Core-proteome analysis of 132 *H. pylori* genomes was performed using the EDGAR database. Essential genes were identified and human and gut microbiota homolog proteins were excluded. The DAH7PS protein involved in the shikimate pathway was selected for the structure-based virtual screening (SBVS) approach. The tertiary structure of the protein was predicted through homology modeling (based on PDB ID: 5UXM). Molecular docking was performed to identify potential inhibitors of DAH7PS among StreptomeDB compounds using the AutoDock Vina tool. Molecular dynamics (MD) simulations assessed the stability of DAH7PS-ligand complexes. The complexes were further evaluated in terms of their binding affinity, Lipinski’s Rule of Five, and ADMET properties.

**Results:**

A total of 54 novel drug targets with desirable properties were identified. DAH7PS was selected for further investigation, and virtual screening of StreptomeDB compounds yielded 36 high-affinity binding of the ligands. Two small molecules, 6,8-Dihydroxyisocoumarin-3-carboxylic acid and Epicatechin, also showed favorable RO5 and ADMET properties. MD simulations confirmed the stability and reliability of DAH7PS-ligand complexes, indicating their potential as inhibitors.

**Conclusion:**

This study identified 54 novel drug targets against *H. pylori*. The DAH7PS protein as a promising drug target was evaluated using a computer-aided drug design. 6,8-Dihydroxyisocoumarin-3-carboxylic acid and Epicatechin demonstrated desirable properties and stable interactions, highlighting their potential to inhibit DAH7PS as an essential protein. Undoubtedly, more experimental validations are needed to advance these findings into practical therapies for treating drug-resistant *H. pylori*.

## 1 Introduction

*Helicobacter pylori* is a Gram-negative, spiral, and microaerophilic bacterium that resides in the human stomach (Suzuki and Moayyedi, 2013). Its discovery in 1982 revolutionized the understanding of gastrointestinal disorders. *Helicobacter pylori* colonizes the stomach mucosa of almost 50% of the global population (Wroblewski et al., 2010) and has an exceptional capacity to adapt to the acidic environment of the stomach (Fong and Fong, 2020). In 2020, *H. pylori*-related disease was the third most common cause of global cancer fatalities, with more than one million new cases of gastric cancer and nearly 800,000 deaths (Sung et al., 2021).

*Helicobacter pylori* is the leading cause of peptic ulcers. However, millions of cases are affected by chronic gastritis, gastric carcinoma, duodenal ulcers, and mucoid-associated lymphoid tissue (MALT) lymphoma due to the infection (Alyahawi et al., 2018; Mahmoud et al., 2021). Asia has a notably high age-standardized rate of gastric cancer because of the widespread prevalence of *H. pylori* infection (Fauzia and Tuan, 2024). Furthermore, the World Health Organization (WHO) has classified *H. pylori* as a class I carcinogen because of its high correlation with gastric cancer (Zhang et al., 2019). Eradicating this pathogen would decrease the occurrence of cancer, leading to a 53% decrease in the incidence in high-risk groups (Chiang et al., 2021). This phenomenon is attributed to reduced inflammation in the gastric mucosa, histological injury, peptic ulcer development, and ulcer recurrence (Wu et al., 2019).

According to the CDC yellow book 2024, asymptomatic infections do not require treatment. The standard treatment for patients with active duodenal or gastric ulcers is bismuth quadruplex therapy, which consists of a proton pump inhibitor (PPI) or H2-blocker, bismuth, metronidazole, and tetracycline. If clarithromycin resistance among *H. pylori* strains is 15% in the region and patients have no prior history of macrolide exposure, clarithromycin triple therapy (PPI + clarithromycin + amoxicillin or metronidazole) is an option (CDC Yellow book, 2024).

Recently, the treatment of *H. pylori* has encountered significant challenges because of antibiotic resistance, leading to treatment failure and recurrence (Hu et al., 2020). Amoxicillin generally exhibits low antibiotic resistance globally, with primary resistance rates of approximately 3% in Asia and 0.4% in Europe. However, in Africa, the resistance rate of *H. pylori* to amoxicillin is significantly higher, averaging 72.6% and reaching up to 100% in certain regions (Lin et al., 2023). Additionally, the majority of WHO regions exhibit a high aggregate prevalence of primary and secondary resistance to metronidazole, clarithromycin, and levofloxacin. The pooled prevalences of resistance to clarithromycin, metronidazole, levofloxacin, amoxicillin, and tetracycline in WHO regions were 15%, 91%, 14%, 38%, and 13%, respectively. Metronidazole exhibited the most prevalence of antibiotic resistance in all regions (Aumpan et al., 2023; Savoldi et al., 2018). Although the prevalence of primary resistance to amoxicillin and tetracycline has remained consistent since 1990, those of clarithromycin, levofloxacin, and metronidazole have been increasing from 1990 to 2022 (Hong et al., 2024; CLSI. Performance Standards for AntimicrobialSusceptibility Testing, 2022).

Moreover, *H. pylori* eradication is further complicated by the emergence of multidrug-resistant (MDR) strains. Under these conditions, the only remaining logical approach is individualized treatment based on antimicrobial susceptibility testing results, which has not been widely adopted. With the evolving and dynamic nature of antibiotic resistance in *H. pylori*, if this situation remains uncontrolled, we may soon confront the risk of running out of effective drug options (Lin et al., 2023). Therefore, it is essential to develop and implement effective strategies to eliminate *H. pylori*. Several drawbacks are associated with conventional drug discovery methods, which impede the efficiency and efficacy of the process. Some of these constraints include high failure rates in identifying viable drug candidates, long timeframes, and high costs (Audah et al., 2019). In contrast, virtual screening methods offer notable advantages over conventional methods. Virtual screening technologies, such as ligand-based and structure-based methods, are pivotal in expediting the drug discovery process by predicting the activity of untested compounds in target drug proteins using computational algorithms (da Silva Rocha et al., 2019; Suzuki et al., 2017). Whole-genome sequencing of bacterial pathogens, high-throughput protein purification, X-ray crystallography, nuclear magnetic resonance (NMR) spectroscopy, and the introduction of bioinformatic methods, molecular docking, and molecular dynamics simulation have led to a better understanding of the structural details of proteins and protein–ligand complexes and drug discovery procedures (Arif et al., 2021; Blundell et al., 2006).

In this study, we aimed to identify novel drug targets against *H. pylori*. Following target identification, a promising therapeutic candidate was selected, and potential inhibitor ligands were screened using a computer-aided drug discovery (CADD) approach. Finally, the feasibility of protein-ligand interactions was assessed using molecular dynamics simulations. The findings of this study might help address the treatment challenges associated with *H. pylori*, which arise due to the increasing rates of antibiotic resistance.

## 2 Materials and methods

### 2.1 Core-proteome analysis

**FIGURE 1:**
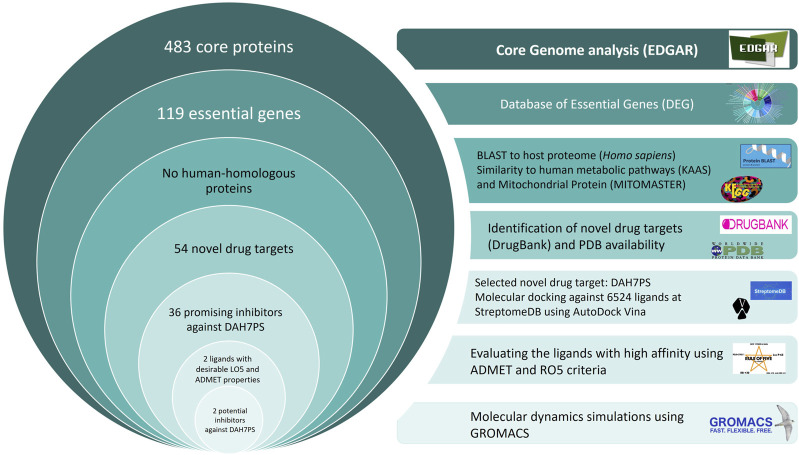
The pipeline for the identification of novel drug targets against *H. pylori*, followed by structure-based virtual screening (SBVS) to identify novel inhibitors against DAH7PS. Two molecules, 6,8-Dihydroxyisocoumarin-3-carboxylic acid and Epicatechin, were introduced as novel promising inhibitors.

The pipeline of the current study for identification of potential drug targets against *H. pylori* has been presented in Figure 1. In the first step, to identify common drug targets among circulating *H. pylori* strains, a total of 132 strains with complete sequence genomes were selected from the EDGAR database (https://edgar3.computational.bio.uni-giessen.de), and the core proteins were extracted in FASTA format.

### 2.2 Identification of novel drug targets

Most antibiotics target critical cellular functions; therefore, key gene products from microbial cells are attractive novel targets for antibacterial drugs (Zhang et al., 2004). Thus, we evaluated the essential role of proteins in the survival of bacteria. For this purpose, the core proteins were compared to the essential gene sequences at database of essential genes (DEG) using BLASTp tool (https://tubic.org/deg/public/index.php).

To minimize any host cross-reactive responses, the essential proteins were blasted against the *Homo sapiens* (taxid: 9606) proteome using BLASTp at NCBI (National Center for Biotechnology Information; https://blast.ncbi.nlm.nih.gov/Blast.cgi). Then, proteins were compared with proteins involved in the metabolic pathways of *H. pylori* and human at the KAAS server (KEGG Automatic Annotation Server). The Kyoto Encyclopedia of Genes and Genomes (KEGG) database includes the metabolic pathways of different organisms and enables the detection of specific metabolic pathways of a particular organism (Moriya et al., 2007). Finally, proteins with any resemblance to those in human metabolic pathways were excluded. Moreover, the remaining proteins were blasted against mitochondrial proteins at the MITOMAP website (https://www.mitomap.org/). These attempts are crucial to prevent the binding of drug targets to human proteins and undesirable cross-reactivities (Kaur et al., 2023).

After retrieving proteins with no similarity to host proteomes, novel drug targets were identified by comparing the proteins to the DrugBank database (https://go.drugbank.com/). This server presents comprehensive data on Food and Drug Administration (FDA)-approved, vet-approved, investigational and experimental drugs, drug targets and interactions as well (Wishart et al., 2018). To fulfill this purpose, proteins with no similarity to the registered targets in the DrugBank database were considered. Next, proteins were submitted to the BLASTp server of NCBI to identify associated protein data bank (PDB) files.

It is well established that antibiotic significantly affect the gut microbiota in terms of diversity and function. Eventually, antibiotics lead to imbalance in gut microbiota (Patangia et al., 2022). To avoid this condition, protein sequences were compared to the proteomes of 83 common commensal strains of the human gut using BLASTp from the GenBank database. This action was taken to select proteins with minimum similarity to the gut microbiota. The gut microbiota strains are listed in Supplementary Data 1.

The subcellular localization of the remaining proteins was then predicted using the PSORTb database (https://www.psort.org/psortb/). Following the described procedure, cytoplasmic proteins were selected as a novel *H. pylori*-targeting drug.

### 2.3 Structure-based virtual screening

#### 2.3.1 Active site conservation and 3D prediction of DAH7PS protein

The 3-deoxy-d-arabino-heptulosonate-7-phosphate synthase (DAH7PS) was selected as a paradigm of potential drug target against *H. pylori*. This protein showed the highest similarity to the protein databank of DAH7PS in *Pseudomonas aeruginosa* (PDB ID: 5UXM). The tertiary structure of DAH7PS of *H. pylori* was predicted by homology modeling using the Swiss-model web server (https://swissmodel.expasy.org/interactive#structure). DAH7PS of *Pseudomonas aeruginosa* (PDB ID: 5UXM) was selected as the template for 3D homology modeling. Then, the predicted tertiary structure was validated using the ProSA-web server (https://prosa.services.came.sbg.ac.at/prosa.php) (Wiederstein and Sippl, 2007) and Ramachandran plots in SWISS-MODEL (https://swissmodel.expasy.org/assess). In addition, the energy minimization of proteins was performed using the GalaxyRefine web-tool (https://galaxy.seoklab.org/cgi-bin/submit.cgi?type=REFINE).

The active site of DAH7PS in *P. aeruginosa* was previously determined via crystal structure analysis by comparing the structure of the protein to that of DAH7PS in other microorganisms and the proximity of allosteric binding sites (Sterritt et al., 2018). Thus, we determined the active site of DAH7PS in *H. pylori* by comparing the protein with its homolog in other microorganisms. The homologous sequences of DAH7PS were investigated in *Pseudomonas aeruginosa* (PDB ID: 5UXM_A), *Mycobacterium tuberculosis* (PDB ID: 2YPO_A), and *Corynebacterium glutamicum* (PDB ID: 5HUC_A) and aligned using Mega X (Kumar et al., 2018). Multiple sequence alignment (MSA) of homologous sequences was performed using the Unipro UGENE software (Okonechnikov et al., 2012).

Next, the conservancy of DAH7PS among different *H. pylori* strains was determined by running of multiple sequence alignment (MSA) of the homologous proteins using the MegaX tool (Kumar et al., 2018). The MSA results were represented in sequence logo format using the WEBLOGO web server (https://weblogo.berkeley.edu/logo.cgi) (Crooks et al., 2004).

#### 2.3.2 Receptor preparation and grid box definition

The protein structure was prepared by assigning bonding orders, adding hydrogen atoms, 0 charges, and removing water molecules. The protein structure was then converted to PDBQT format for molecular docking analysis. The grid box was defined as Center_X = 21.414 Å, Center_Y = 16.331 Å, and Center_Z = 24.558 Å grid points, and the grid spacing was considered 1.000 Å. The grid box is defined over the protein’s structure that focuses the docking simulation on the active site of the protein. We concluded hydrogen-donor residues in the active site could be associated with hydrogen bonds between protein-ligand complex. Thus, we considered a grid box enclosed all hydrogen-donor residues.

#### 2.3.3 Ligand preparation

Structure-based virtual screening (SBVS) as *in silico* process is a widely recognized approach for drug development. This strategy detects selective inhibitors for the binding site of the targeted protein (Li et al., 2017). Secondary metabolites originate from *Streptomyces* spp. StreptomeDB contains 6524 ligands were considered as a library of small molecules (Moumbock et al., 2020). Thus, we aimed to explore novel inhibitors of DAH7PS using the StreptomeDB databases. All compounds were downloaded in SDF format and then converted to PDBQT format using OpenBabel software. Meanwhile, energy minimization was performed on all ligands using OpenBabel with the MMFF94 force field, and the ligands were exported into PDBQT format (O’Boyle et al., 2011).

#### 2.3.4 Molecular docking of DAH7PS with ligands

The prepared ligands were docked in the active sites of DAH7PSs with a defined grid box using AutoDock Vina software (Eberhardt et al., 2021). The docking results were sorted by the descending binding affinity. Subsequently, ligands with a binding affinity ≤ −13 kcal/mol were selected and evaluated based on Lipinski’s Rule of Five (RO5) and ADMET properties. Rigid and flexible dockings were performed once again for selected molecules. According to RO5, a drug is more likely to have good absorption and permeation if it has 5 or fewer hydrogen bond donors, 10 or fewer hydrogen bond acceptors, a molecular weight of 500 kDa or less, and a calculated Log P (CLog P) of 5 or below (Benet et al., 2016). The ADMET index predicts the Absorption, Distribution, Metabolism, Excretion, and Toxicity of a compound (Guan et al., 2019). The interacting residues were visualized using the BIOVIA Discovery Studio Visualizer (Studio, 2008). Finally, the APBS Electrostatics plugin in PyMOL software was used to visualize the Electrostatic charge distribution of the most promising complexes (Lerner and Carlson, 2006).

#### 2.3.5 Molecular dynamics simulations

To evaluate the stability of interactions within the DAH7PS-ligand complexes, molecular dynamics (MD) simulations were carried out using the GROMACS 2022 simulation package and the CHARMM force field (Paul Bauer, 2022). First, the structural system was placed in an octahedron box with 1.2-nm spacing from all edges (Jørgensen and Reisfeld, 1983). The topology parameters of the protein structures were generated using the GROMACS software suite. For each ligand, the topology and configuration parameters were obtained via the SwissParam server (http://www.swissparam.ch/). Subsequently, the simulation box was filled with TIP3 water molecules. To neutralize the system, appropriate quantities of Na^+^ and Cl^−^ ions were added. Three-dimensional periodic boundary conditions were applied to the entire system.

MD simulations began with a two-stage energy minimization process. First, the systems were equilibrated under NVT (constant Number of particles, Volume, and Temperature) conditions at 300 K for 100 ps. This was followed by an NPT (constant Number of particles, Pressure, and Temperature) equilibration phase lasting 1,000 ps, in which the Parrinello-Rahman barostat was used to maintain the temperature at 300 K and the pressure at 1.0 bar.

Long-range electrostatic interactions were calculated using the Particle Mesh Ewald (PME) method with a cutoff distance of 10 Å. Van der Waals (VDW) interactions were determined using a 1-nm cut-off. To ensure the stability of hydrogen bonds, the LINCS algorithm was applied to constrain all bonds involving hydrogen atoms. The final stage of the MD simulation involved a 100-ns run without restraints. After the necessary equilibration steps, the first 10 ns were considered as the equilibrium period, resulting in a 40-ns simulation with 2-fs time steps. Following equilibration, 100-ns production runs were executed for the docked complexes. To evaluate the stability of the protein-ligand complexes, essential metrics such as root mean square deviation (RMSD), root mean square fluctuation (RMSF), and the radius of gyration (Rg) were calculated (Jahantigh et al., 2022). RMSD, in particular, was used to assess conformational changes in the protein during MD simulation, with lower RMSD values indicating greater stability of the docked complex (Cardoso and Mendanha, 2021). RMSF was used to measure the fluctuations of individual residues within the complex, with higher RMSF values indicating greater flexibility of the protein’s regions (Ghahremanian et al., 2022). Additionally, Rg was calculated to assess the compactness and folding of the docked complexes. Lower Rg values indicate a more rigid and compact structure (Jiang et al., 2019; Parida et al., 2020).

## 3 Results

### 3.1 Lis of new drug targets against *Helicobacter pylori*

Core-genome analysis identified 483 core genes within a dataset of 132 *H. pylori* strains. Out of 483 proteins, 119 with high similarity to essential DEG proteins were identified. By comparing 119 essential proteins to the host proteome, 55 proteins were excluded to prevent cross-reaction with *H. sapiens*. None of the remaining 64 proteins resembled human metabolic pathways at the KAAS server and mitochondrial proteins at the MITOMASTER database.

Proteins were investigated in the drug target of the DrugBank database. Out of 64 proteins, 10 were previously targeted by FDA-approved experimental and investigational drugs, and 54 potential novel drug targets were identified. Among these novel drug targets, 31 proteins were similar to a protein available in the PDB file in the PDB database of NCBI. The subcellular localization of the proteins was predicted for 21 cytoplasmic membrane proteins, 16 unknown proteins, 12 cytoplasmic membrane proteins, 2 flagellar proteins, 2 OMPs, and 1 periplasmic protein. Among the remaining proteins, four showed no similarity to the human gut microbiota: 3-deoxy-7-phosphoheptulonate synthase class II (WP_015085380.1), LPP20 lipoprotein (WP_000795968.1), ComF family protein (WP_015086367.1), and FolB domain-containing protein (WP_015086400.1). The 54 potential new drug targets are detailed in Table 1.

**TABLE 1:** The shortlist of novel drug targets from *Helicobacter pylori*.

No.	Protein	Accession number	Protein target	PDB target	Coverage	Identity	Subcellular localization	Gut microbiota coverage	Gut microbiota Identity
1	3-deoxy-7-phosphoheptulonate synthase class II	WP_015085380.1	Chain A, Phospho-2-dehydro-3-deoxyheptonate aldolase [*Pseudomonas aeruginosa* PAO1]	5UXM_A	99%	61.02%	Cytoplasmic	—	—
2	Cytochrome-c oxidase, cbb3-type subunit II	WP_000490769.1	Chain B, Cytochrome c oxidase, cbb3-type, subunit O [*Stutzerimonas stutzeri*]	3MK7_B	70%	50.30%	Cytoplasmic	98%	47.67%
3	Cytochrome c oxidase, cbb3-type, CcoQ subunit	WP_002205208.1	—	—	—	—	Unknown	65%	22.92%
4	DUF4006 family protein	WP_000670512.1	—	—	—	—	Unknown	—	—
5	Elongation factor P	WP_245060244.1	Chain A, Elongation factor P [*Acinetobacter baumannii*]	5J3B_A	95%	47.25%	Cytoplasmic	97%	49.46%
6	Hypothetical protein	WP_015085488.1	Chain A, Indole-3-glycerol phosphate synthase [*Campylobacter jejuni* subsp. *jejuni* NCTC 11168 = ATCC 700819]	6BMA_A	95%	27.49%	Cytoplasmic	30%	32.76%
7	DUF4149 domain-containing protein	WP_015085499.1	—	—	—	—	Cytoplasm membrane	—	—
8	50S ribosomal protein L21	WP_101001314.1	Chain R, 50S ribosomal protein L21 [*Escherichia coli* K-12]	2J28_R	95%	47.10%	Cytoplasmic	97%	45.10%
9	Flagellar motor switch protein FliG	WP_000201862.1	Chain A, Flagellar motor switch protein [*Helicobacter pylori*]	3USW_A	75%	98.84%	Flagella	95%	40.06%
10	Outer membrane protein	WP_041200001.1	—	—	—	—	Outer Membrane	—	—
11	Response regulator-like transcription factor HsrA	WP_001264984.1	Chain A, Putative TRANSCRIPTIONAL REGULATOR [*Helicobacter pylori* J99]	2HQR_A	99%	100%	Cytoplasmic	95%	28.05%
12	30S ribosomal protein S21	WP_001117778.1	Chain u, 30S ribosomal protein S21 [*Escherichia coli*]	3J9Y_u	84%	48.33%	Unknown	84%	48.33%
13	Hypothetical protein	WP_000495089.1	—	—	—	—	Unknown	—	—
14	Endolytic transglycosylase MltG	WP_015085707.1	Chain A, Predicted aminodeoxychorismate lyase [*Escherichia coli* K-12]	2R1F_A	55%	30.73%	Cytoplasmic	50%	41.62%
15	Efflux RND transporter outer membrane subunit HefA	WP_015085721.1	—	—	—	—	Outer membrane	—	—
16	uracil-DNA glycosylase	WP_041200012.1	—	—	—	—	Unknown	—	—
17	DUF2603 domain-containing protein	WP_000413468.1	—	—	—	—	Cytoplasmic	—	—
18	Phosphatidylglycerophosphatase A	WP_015085822.1	—	—	—	—	Cytoplasmic Membrane		37.18%
19	Hypothetical protein	WP_041200020.1	—	—	—	—	Unknown	—	—
20	D-glycero-beta-D-manno-heptose 1,7-bisphosphate 7-phosphatase	WP_015085922.1	Chain A, D,D-heptose 1,7-bisphosphate phosphatase [*Escherichia coli* K-12]	3L8E_A	90%	40.96%	Cytoplasmic	86%	49.06%
21	HypC/HybG/HupF family hydrogenase formation chaperone	WP_180431173.1	Chain A, Hydrogenase assembly chaperone hypC/hupF [*Shewanella oneidensis* MR-1]	3D3R_A	94%	50%	Unknown	87%	38.57%
22	Hypothetical protein	WP_015085965.1	—	—	—	—	Unknown	—	—
23	Zinc metalloprotease HtpX	WP_162470558.1	Chain A, Probable protease htpX homolog [*Vibrio parahaemolyticus* RIMD 2210633]	3CQB_A	25%	35.71%	Cytoplasmic Membrane	96%	40.63%
24	Hypothetical protein	WP_015086008.1	—	—	—	—	Periplasmic	—	—
25	Hypothetical protein	WP_000555362.1	—	—	—	—	Unknown	—	—
26	ExbD/TolR family protein	WP_001105106.1	Chain Y, Biopolymer transport protein ExbD [*Escherichia coli* K-12]	6TYI_Y	88%	31.36%	Cytoplasmic Membrane	88%	29.66%
27	MotA/TolQ/ExbB proton channel family protein	WP_015086112.1	Chain A, Tol-Pal system protein TolQ [*Escherichia coli* K-12]	8ODT_A	46%	44.32%	Cytoplasmic Membrane	86%	29.79%
28	F0F1 ATP synthase subunit delta	WP_015086113.1	—	—	—	—	Cytoplasmic	—	—
29	F0F1 ATP synthase subunit B	WP_001027653.1	—	—	—	—	Unknown	—	—
30	KH domain-containing protein	WP_015086123.1	—	—	—	—	Unknown	56%	33.85%
31	rRNA maturation RNase YbeY	WP_041200032.1	Chain A, Hypothetical protein AQ_1354 [*Aquifex aeolicus*]	1OZ9_A	66%	46.39%	Cytoplasmic	80%	40.94%
32	F0F1 ATP synthase subunit C	WP_015086170.1	Chain A, ATP SYNTHASE SUBUNIT C [*Fusobacterium nucleatum*]	3ZK1_A	76%	38.37%	Cytoplasmic Membrane	59%	38.10%
33	RDD family protein	WP_015086174.1	—	—	—	—	Cytoplasmic Membrane	—	—
34	NADH-quinone oxidoreductase subunit NuoK	WP_000579762.1	Chain G, NADH-QUINONE OXIDOREDUCTASE SUBUNIT K [*Escherichia coli* BL21 (DE3)]	3RKO_G	99%	40%	Cytoplasmic Membrane	98%	43.27%
35	50S ribosomal protein L36	WP_000868339.1	Chain 4, 50S ribosomal protein L36 [*Staphylococcus aureus* subsp. *aureus* NCTC 8325]	4WCE_4	97%	72.97%	Cytoplasmic	97%	78.38%
36	50S ribosomal protein L15	WP_001870913.1	Chain l, 50S ribosomal protein L15 [*Escherichia coli* BL21 (DE3)]	6QDW_l	82%	45.05%	Cytoplasmic	99%	42.22%
37	50S ribosomal protein L18	WP_000991853.1	Chain AQ, 50S ribosomal protein L18 [*Borreliella burgdorferi* B31]	8FMW_AQ	78%	50.53%	Cytoplasmic	78%	50.53%
38	50S ribosomal protein L6	WP_015086235.1	Chain G, 50S ribosomal protein L6 [*Pseudomonas aeruginosa* PAO1]	7UNR_G	97%	49.71%	Cytoplasmic	98%	49.44%
39	50S ribosomal protein L24	WP_000834238.1	Chain S, 50S ribosomal protein L24 [*Staphylococcus aureus*]	7ASM_S	90%	58.21%	Cytoplasmic	89%	56.06%
40	50S ribosomal protein L23	WP_000763613.1	—	—	—	—	Unknown	91%	35.56%
41	TonB system transport protein ExbD	WP_000836362.1	Chain F, Biopolymer transport protein ExbD [*Serratia marcescens*]	7AJQ_F	84%	38.05%	Cytoplasmic Membrane	84%	38.05%
42	SH3 domain-containing protein	WP_01508622.1	—	—	—	—	Unknown	—	—
43	Hypothetical protein	WP_015086273.1	—	—	—	—	Cytoplasmic	—	—
44	NADPH-dependent 7-cyano-7-deazaguanine reductase QueF	WP_015086313.1	Chain A, NADPH-dependent 7-cyano-7-deazaguanine reductase [*Bacillus subtilis* subsp. *subtilis* str. 168]	4F8B_A	90%	57.04%	Cytoplasmic	97%	57.93%
45	Ribosome silencing factor	WP_001042231.1	Chain 6, Ribosomal silencing factor RsfS [*Escherichia coli* K-12]	7BL2_6	76%	28.41%	Cytoplasmic	76%	36.96%
46	Carbon storage regulator	WP_000906444.1	Chain A, Carbon storage regulator [*Bacillus subtilis*]	1T3O_A	77%	45.00%	Unknown	92%	43.66%
47	Membrane protein insertion efficiency factor YidD	WP_001245491.1	—	—	—	—	Cytoplasmic Membrane	55%	43.08%
48	LPP20 lipoprotein	WP_000795968.1	Chain A, LPP20 lipoprotein [*Helicobacter pylori* J99]	5OK8_A	99%	99.43%	Unknown	—	—
49	ComF family protein	WP_015086367.1	Chain A, *Helicobacter pylori* ComF fused to an artificial alphaREP crystallization helper (named B2) [synthetic construct]	7P0H_A	99%	95.29%	Cytoplasmic	—	—
50	Protoporphyrinogen oxidase HemJ	WP_000395127.1	—	—	—	—	Cytoplasmic Membrane	—	—
51	FolB domain-containing protein	WP_015086400.1	Chain A, Dihydroneopterin aldolase [*Helicobacter pylori* G27]	8EVK_A	99%	89.74%	Cytoplasmic	—	—
52	Hypothetical protein	WP_025222353.1	—	—	—	—	Cytoplasmic Membrane	—	—
53	Flagellar basal body rod protein FlgC	WP_000480077.1	Chain V, Flagellar basal-body rod protein FlgC [*Salmonella enterica* subsp. *enterica* serovar Typhi]	7BIN_V	92%	31.33%	Flagella	95%	42.95%
54	Septal ring lytic transglycosylase RlpA family protein	WP_000521812.1	Chain A, PA4485 [*Pseudomonas aeruginosa* PAO1]	4AVR_A	27%	51.14%	Unknown	28%	60.44%

### 3.2 *In silico* characterization of DAH7PS protein

After careful evaluations, DAH7PS (WP_015085380.1) was selected as a potential *H. pylori*-targeting drug. This enzyme is a part of the shikimate pathway and is present in microorganisms but not in mammals. This pathway is essential for the biosynthesis of important aromatic compounds, including phenylalanine (Phe), tyrosine (Tyr), and tryptophan (Trp). DAH7PS facilitates an aldol-like condensation reaction between phosphoenolpyruvate (PEP) and erythrose 4-phosphate (E4P), resulting in the formation of 3-deoxy-D-arabino-heptulosonate 7-phosphate (DAH7P) (Sterritt et al., 2018). The absence of this pathway and the enzymes in mammals make them possible targets for the development of antibiotics without any cross-reactive reactions in the host (Frlan, 2022).

**FIGURE 2:**
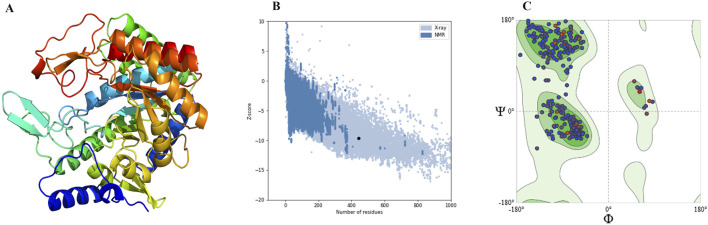
**(A)** Tertiary structure of DAH7PS of *H. pylori* predicted through homology modeling. **(B)** Validation of the predicted 3D structure using ProSA-Web analysis, showing a Z-score = −9.64. **(C)** Ramachandran plot of the predicted structure of DAH7PS, showing that 97.06% of the residues were located in the favored region.

**FIGURE 3:**
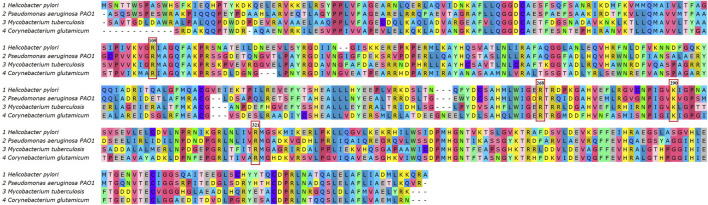
Multiple sequence alignment of the DAH7PS protein in *H. pylori*, *Pseudomonas aeruginosa*, *Mycobacterium tuberculosis*, and *C. glutamicum* showed high conserved DAH7PS expression. The H-donor residues of active site including Arg109, Arg 268, Lys 290, and Arg 321 are indicated in red boxes.

**FIGURE 4:**
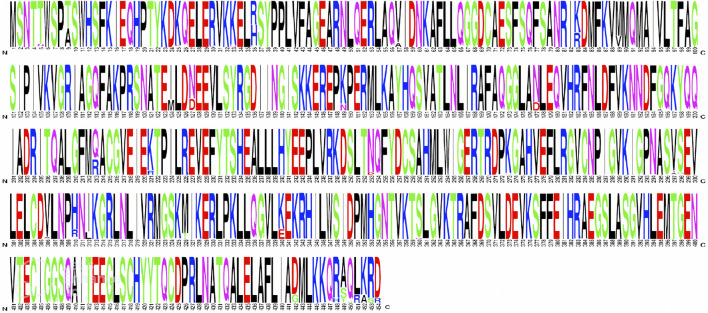
Multiple sequence alignment of the DAH7PS protein among 132 *H. pylori* strains visualized using the WEBLOGO web server.

The 3D structure of DAH7PS from *H. pylori* was predicted by homology modeling. The tertiary structure, ProSA-web plot, and Ramachandran plot are presented in Figure 2. ProSA-web analysis showed a Z-score = −9.64. The QMEAN global score of the predicted tertiary structure was 0.83, which confirms the high quality of the PDB file. The Ramachandran plot demonstrates that 97.06% of the residues were located in the favored region. Multiple sequence alignment of the DAH7PS protein showed high conserved active site residues in *H. pylori*, *P. aeruginosa*, M. *tuberculosis*, and *C. glutamicum*. The MSA and hydrogen donor residues in the active site (Arg109, Arg 268, Lys 290, Arg 321) are shown in Figure 3. Moreover, the MSA of DAH7PS was conserved among *H. pylori* strains. See Figure 4.

### 3.3 Virtual screening results

**FIGURE 5:**
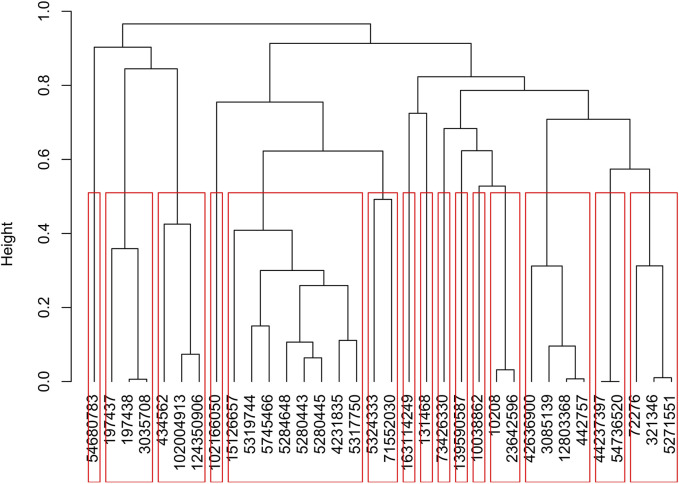
Hierarchal clustering (based on the Tanimoto coefficient) classified the 36 ligands with the highest affinity (≤−13 kcal/mol) to DAH7PS into 15 clusters.

The molecular dockings of DAH7PS against 6524 compounds at StreptomeDB resulted in the identification of 36 ligands with the highest affinity (≤−13 kcal/mol). Based on the Tanimoto coefficient, hierarchal clustering of the ligands identified 15 clusters among the 36 ligands (Figure 5). All compounds exhibited desirable RO5 activities (Supplementary Data 2).

2′,5′-Dimethoxyflavone, 3′,4′,7-Trihydroxyisoflavone, 4′-Hydroxy-5,7-Dimethoxyflavanone, 5-Hydroxy-7,4′-Dimethoxyflavanone, AC1NT4ZV, Apiigenin, Bhimamycin A, Bhimamycin I, Glycitein, Furthermore, Nanaomycin alpha had Log Papp >1 × 10^−6^ cm/s. Compounds with a Log Papp: 1-10 × 10^−6^ cm/s are classified as moderately absorption in the human intestine (Kus et al., 2023). Other ligands exhibited lower intestinal absorption.

Austramide, Bhimamycin A, Taxifolin, and N-(4,7-Dihydroxy-8-methyl-2-oxo-2H-chromen-3-yl)-1H-pyrrole-2-carboxamide had a VD_ss_ > 0.7 L/kg, indicating a high distribution of these compounds in tissues (Lombardo et al., 2021).

Among the studied compounds, only 5-Hydroxy-7,4′-dimethoxyflavanone had a Log BB ≥ 0.3 that could pass the blood–brain barrier (Kunwittaya et al., 2013), while 2′,5′-dimethoxyflavone and 7,4′-dihydroxy-8-methoxy-isoflavone showed Log PS > 0.2, demonstrating their capability to penetrate the CNS (Kalhor et al., 2023).

2-Acetyl-1,8-dihydroxy-3-methylanthraquinone, 3-Indolylcarbonyl, alpha-L-rhamnopyranoside, 6,8-Dihydroxyisocoumarin-3-carboxylic acid, Antimycin, Bhimamycin A, CHEMBL492618, Epicatechin, Nanaomycin A, Fluostatin P, Taxifolin, Nanaomycin C, Nanaomycin alpha, Nikkomycin So (X), Nikkomycin So (Z), Nikkomycin S (X), Octoketide 4b, RK 1441A, SEK4b, and cyclo (3-Hydroxy-L-Pro-L-Tyr) are neither substrates nor inhibitors of CYP enzymes. Almost all selected ligands, except 2′,5′-dimethoxyflavone, were not the substrate of Renal OCT2, indicating that the compounds are not excreted through the kidneys.

5,7,4′-trihydroxy-3′-methoxyisoflavone, 6,8-Dihydroxyisocoumarin-3-carboxylic acid, 7,4′-Dihydroxy-8-methoxy-isoflavone, AC1NT4ZV, Antimycin, Austramide, CHEMBL492618, Epicatechin, Fluostatin P, Taxifolin, Luteolin, Naomycin C, Naomycin alpha, SEK4b, and cyclo(D)-tran-4-OH-Pro-(D)-Phe exhibited no toxicity in the following tests: Oral rat acute and chronic toxicity, *Tetrahymena pyriformis* toxicity, minnow toxicity, hepatotoxicity, or skin sensitization. Additionally, they were not inhibitors of hERG I and II.

**FIGURE 6:**
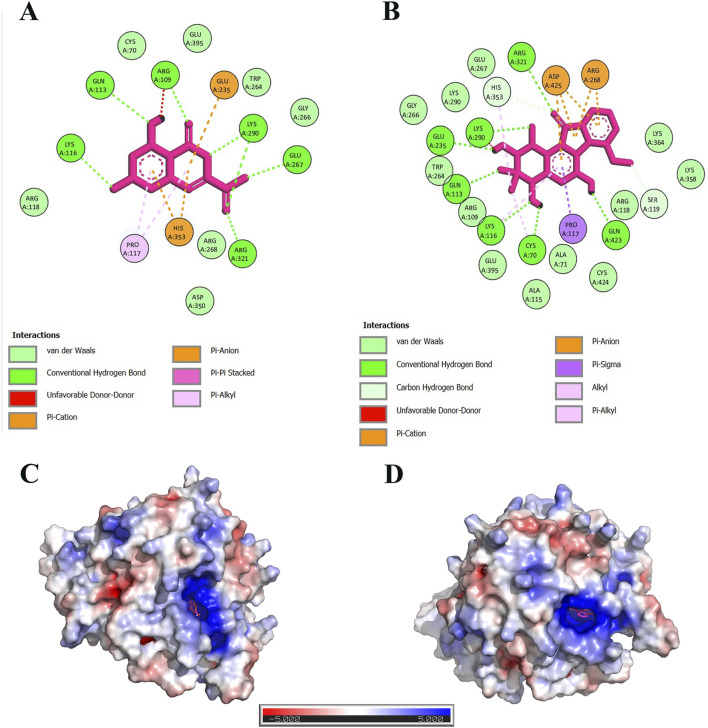
The interactions of 6,8-Dihydroxyisocoumarin-3-carboxylic acid **(A)** and Epicatechin **(B)** with DAH7PS protein. The tertiary structure of the DAH7PS protein and its APBS (Adaptive Poisson-Boltzmann Solver) electrostatic interactions with the ligands are shown in **(D)** and **(C)** depictions. Data highlight that the pocket size has a highly positive charge.

The docked complexes were visualized in the Discovery Studio software. Seven hydrogen bonds were formed between the 6,8-Dihydroxyisocoumarin-3-carboxylic acid and Lys 116, Gln 113, Arg 109, Lys 290, Glu 267, and Arg 321 residues of DAH7PS. Moreover, two pi-Alkyl bonds with Pro 117, one pi-pi stacked with Arg 109, one cationic bond with His 353, and one anionic bond with Glu 235 were observed (Figure 6A).

Epicatechin formed seven hydrogen bonds with Arg 321, Lys 290, Glu 235, Gln 113, Lys 116, Cys 70, and Gln 423 residues in DAH7PS. Furthermore, two Van der Waals residues with His 353 and Ser 119, three alkyl bonds with His 353, Lys 116, Cys 70, one cationic bond with Arg 268, one anionic bond with Asp 425, and one pi-sigma with Pro 117 (Figure 6B).

Electrostatic charge distribution of 6,8-Dihydroxyisocoumarin-3-carboxylic acid-DAH7PS, and Epicatechin-DAH7PS complexes are presented in Figures 6C, D, respectively. Both complexes were positively charged around the active site of the protein. Thus, small molecules with negative electrostatic charges have a stronger interaction with this positive cavity.

### 3.4 The results of molecular dynamics simulations

**FIGURE 7:**
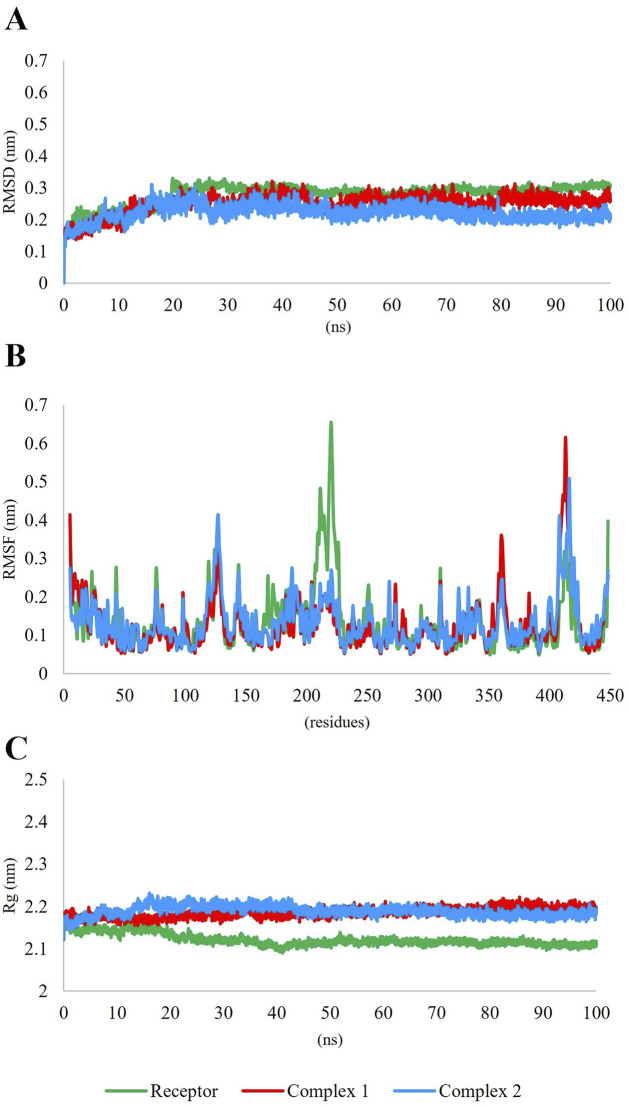
Molecular dynamic (MD) simulation data of DAH7PS-ligand complexes. Complex 1 shows 6,8-dihydroxyisocoumarin-3-carboxylic acid and complex 2 shows Epicatechin with DAH7PS. **(A)** The RMSD plot of both complexes fluctuates between 0.1 and 0.3, indicating the stability of the complexes. **(B)** the RMSF of both complexes fluctuates between 0 and 0.7. **(C)** The Rg. plots of both complexes were slightly higher than that of the sole protein.

Thus, 6,8-Dihydroxyisocoumarin-3-carboxylic acid, and epicatechin, which showed desirable RO5 and ADMET properties, were selected as the most promising inhibitors against DAH7PS in MD simulations. RMSD, RMSF, and Rg, which are fundamental metrics, were employed in MD simulations to evaluate the stability, flexibility, and compactness of biomolecular systems (Nipun et al., 2021). The MD simulation plots of the two complexes are presented in Figure 7. Complex 1 represents the complex of DAH7PS and 6,8-dihydroxyisocoumarin-3-carboxylic acid, and complex 2 represents the complex of DAH7PS and Epicatechin. RMSD measures the deviation of atomic positions over time, indicating structural changes in the system, while RMSF provides insights into the flexibility of individual residues within a protein. The RMSD values of both complexes fluctuated between 0.1 and 0.3, indicating the stability of the complexes (Figure 7A), and the RMSF values of both complexes varied between 0 and 0.7 (Figure 7B). On the other hand, Rg provides information about the overall compactness of a biomolecule, thereby aiding in understanding its global shape and conformation (Nipun et al., 2021). The Rg plots were slightly higher in DAH7PS-ligand complexes than for the sole protein (Figure 7C). These parameters play a crucial role in MD studies aimed at assessing the behavior of ligand-receptor complexes, protein-ligand interactions, and the stability of compounds (Erol, 2023).

## 4 Discussion

*Helicobacter pylori* is a significant bacterium in healthcare systems because of its widespread prevalence and association with various gastrointestinal diseases and malignancy. Approximately half of the global population is infected with *H. pylori* (Elbehiry et al., 2023; Cover and Blaser, 2009). The treatment of *H. pylori* has become challenging because of increasing antibiotic resistance. Resistance to antibiotics has increased due to the widespread use of antibiotics, which renders less effectiveness of standard treatment regimens. Resistance rates vary geographically but are generally rising, complicating eradication efforts (Elbehiry et al., 2023). Antimicrobial resistance to commonly used antibiotics such as clarithromycin, metronidazole, and levofloxacin has been particularly problematic, necessitating the development of new treatment strategies.

CADD techniques like virtual screening can rapidly evaluate large chemical libraries to identify promising lead compounds that inhibit bacterial targets. Machine learning models trained on antimicrobial activity data can predict potency and help prioritize compounds for synthesis and testing (Melo et al., 2021; da Silva et al., 2022; Jukič and Bren, 2022). This accelerates the lead discovery process significantly compared to traditional trial-and-error screening. Moreover, *in silico* approaches allow a computational prediction of ADMET (absorption, distribution, metabolism, excretion, toxicity) properties to optimize drug-likeness early in the discovery process. This strategy reduces late-stage failure of compounds due to poor pharmacokinetics. (Ameji et al., 2024). Modeling atomic-level interactions facilitates the identification of inhibitors that are less susceptible to common resistance mechanisms like target mutations. This increases the likelihood of discovering antibiotics that remain effective against resistant strains (Almihyawi et al., 2022).

Several antibiotics discovered using CADD approaches have progressed to clinical trials or regulatory approval. Solithromycin, a novel fluoroketolide, was designed using computational modeling and docking to overcome resistance to earlier macrolides. It showed potent activity against drug-resistant pneumonia in phase 3 trials. Gepotidacin, a novel triazaacenaphthylene bacterial topoisomerase inhibitor, was identified through virtual screening and optimization. The efficacy of the agent against uncomplicated urinary tract infections caused by resistant Enterobacterales was demonstrated in a phase 2 trial study (Melo et al., 2021).

Although CADD has greatly accelerated antibiotic discovery, significant challenges remain in translating promising *in silico* hits into clinically useful drugs. Integrating computational approaches with biophysical simulations, machine learning, and experimental data is key to maximizing their impact. Overall, such approaches have become indispensable tools for combating antibiotic resistance, complementing and enhancing traditional discovery methods (Almihyawi et al., 2022).

In the current study, we performed a subtractive proteome analysis to identify the most promising drug targets against *H. pylori*. For this purpose, several important properties were considered. For example, core proteins conserved across circulating *H. pylori* strains are ideal drug targets because they ensure broad-spectrum efficacy. Targeting these proteins increases the likelihood that a developed drug will be effective against various *H. pylori* strains, thereby reducing the risk of treatment failure due to strain-specific variations. In addition, essential genes are critical for bacterial survival. Targeting essential gene products increases the likelihood of developing potent antibacterial drugs. If an essential gene product is inhibited, it can lead to bacterial death or severe growth inhibition, making it an effective therapeutic strategy (Patil et al., 2013; Luo et al., 2015). In addition to improving the safety profile of the drug, proteins unique to *H. pylori* that specifically affect the pathogen without interfering with human cellular processes were targeted. Mitochondria, being evolutionarily related to bacteria, often share similarities in their protein structures (Boguszewska et al., 2020). Ensuring that target proteins are not similar to mitochondrial proteins helps avoid potential off-target effects on human mitochondria, which could lead to cellular toxicity. The availability of protein structures in the PDB is valuable for structure-based virtual screening. Having detailed structural information allows for *in silico* modeling and rational drug design, potentially accelerating the drug discovery process and improving the likelihood of developing effective inhibitors. These properties, when combined, help identify drug targets that are likely to be effective against *H. pylori*, safe for human use, and amenable to rational drug design approaches for developing successful treatments.

Taken together, genomic subtraction identified DAH7PS as one of the novel and promising drug targets against this pathogen. This enzyme plays a critical role in the shikimate pathway (Jiao et al., 2020). Because *H. pylori* relies on this pathway to synthesize amino acids that are not available in the host, targeting DAH7PS may effectively disrupt its metabolic processes, leading to bacterial growth inhibition or death (Divyashri et al., 2021). Conversely, the shikimate pathway is absent in mammals, making it an attractive target for antibiotic development. This specificity reduces the risk of off-target effects in human cells, thereby increasing the safety profile of potential drugs (Han et al., 2006).

The shikimate pathway plays a fundamental role in the survival and virulence of bacteria through several critical functions. This pathway is essential for the biosynthesis of aromatic amino acids, which are indispensable for protein production and bacterial growth. Additionally, the shikimate pathway leads to the formation of chorismate, a key precursor for the synthesis of vital compounds such as folic acid and siderophore. These metabolites are crucial for bacterial metabolism and their ability to acquire iron, a necessary element for survival in host environments. Studies have demonstrated that interference with this pathway can significantly reduce bacterial growth and virulence, highlighting its pivotal role in the pathogenic life cycle (Frlan, 2022).

Because the shikimate pathway is conserved among many pathogenic bacteria, DAH7PS inhibitors may also be effective against other bacterial pathogens, providing a broader therapeutic application. This pathway has been considered a potential drug target for various pathogens, including *M. tuberculosis* and *Acinetobacter baumannii* (Nunes et al., 2020; Shil et al., 2023).

DAH7PS showed a positive electrostatic charge distribution at its active site. Positive electrostatic charges on proteins attract negatively charged ligands, enhancing binding affinity due to Electrostatic interaction between ionic species of the opposite charges. Charged residues provide specific and highly stable binding by forming strong interactions with negatively charged ligand groups (Zhou and Pang, 2018).

SBVS resulted in the identification of 36 potential inhibitors against DAH7PS. Among them, 6,8-Dihydroxyisocoumarin-3-carboxylic acid and Epicatechin, which possess desirable ADMET properties, were selected for MD simulation studies. MD simulation has revolutionized drug discovery by providing atomic-level insights into drug-target interactions and enhancing various stages of the drug development process. It can reveal hidden binding pockets on protein targets that are not obvious in static crystal structures. This allows the discovery of novel binding sites and allosteric modulators. It generates ensembles of receptor conformations that can be used in virtual screening, allowing for a more dynamic and realistic representation of the target, which enhances the accuracy of docking and scoring compared to rigid receptor structures. Long timescale MD simulations can provide estimates of drug binding and unbinding rates, which are important for understanding drug efficacy and residence time. Unlike static docking, MD simulations account for protein flexibility and entropic effects, providing a more accurate representation of the thermodynamics of drug binding (De Vivo et al., 2016; Durrant and McCammon, 2011). With ongoing improvements in algorithms and computing power, MD simulations are likely to play an increasingly important role in CADD. Our analysis showed that 6,8-Dihydroxyisocoumarin-3-carboxylic acid and Epicatechin both passed the RO5 rule, which demonstrates the oral bioavailability of these compounds in humans (Roskoski, 2019).

Epicatechin is a polyphenolic derivative of green tea with anti-inflammatory and antioxidant effects (Shukla et al., 2019; Ongnok et al., 2020). Moreover, several studies have reported the antibacterial effects of this compound on *Stenotrophomonas maltophilia*, *A. baumannii*, and *Staphylococcus aureus* (Betts et al., 2011; Guo et al., 2022). Similarly, it has shown promising antibacterial effects against drug-resistant *H. pylori* strains (Escandón et al., 2016; Yanagawa et al., 2003). One possible mechanism by which catechins exert antibacterial effects is perturbation of the bacterial membrane by targeting phospholipids. However, its mechanism of action has not been fully understood (Escandón et al., 2016; Yanagawa et al., 2003). Our study might shed light on a new aspect of its mechanism of action by targeting the DAH7PS enzyme in the shikimate pathway.

6,8-Dihydroxyisocoumarin-3-carboxylic acid is a coumarin derivative. Coumarin compounds exhibit possess antioxidant, anti-inflammatory, anti-cancer, antimicrobial, anti-HIV, and anti-tuberculosis properties (Li et al., 2024). Hydroxylated coumarins such as 7-hydroxy-4-methylcoumarin, 6,7-dihydroxy-4-methylcoumarin, 6-hydroxy-7-methoxy-4-methylcoumarin, and 5,7-dihydroxy cyclopentanocoumarin have shown anti-*H. pylori* effects as strong as metronidazole (Kawase et al., 2003). Similarly, another study measured the MIC of 24 coumarin derivatives against *H. pylori* and reported that the majority of derivatives indicated an MIC of 10–40 mg/mL. Moreover, they demonstrated potential urease inhibitory effects (Jadhav et al., 2013).

It is noteworthy that several previous studies have identified novel drug targets and inhibitors for treating *H. pylori*. Gonzalez et al. targeted HsrA, and seven natural flavonoids were identified as potential inhibitors of this protein (González et al., 2019). Similarly, HsrA was identified as a shortlisted drug target in our study. In another study, Divyashri G et al. aimed to identify potential inhibitors of *H. pylori* in mango ginger by conducting molecular docking of 130 compounds against selected drug targets. The findings revealed that mango ginger compounds exhibited good binding affinity toward shikimate kinase and type II dehydroquinase through interactions like hydrogen bonds and salt bridges (Divyashri et al., 2021).

## 5 Conclusion

In conclusion, this study focused on identifying novel therapeutic agents for targeting drug-resistant *H. pylori* infections. Using subtractive proteomics, 54 new drug targets were identified, with DAH7PS emerging as a particularly promising candidate. Two potential inhibitors, 6,8-Dihydroxyisocoumarin-3-carboxylic acid and Epicatechin, were identified, both of which demonstrated favorable RO5 and ADMET properties. MD simulations confirmed the stability and reliability of the DAH7PS-ligand complexes, thereby underscoring their potential effectiveness. However, it is crucial to integrate computational predictions with experimental validation to advance these promising compounds into practical applications. Our findings provide valuable insights into the development of new targeted therapies against *H. pylori* in future studies.

## Data Availability

The original contributions presented in the study are included in the article/Supplementary Material, further inquiries can be directed to the corresponding author.

## Abbreviations

ADMET, Absorption, Distribution, Metabolism, Excretion, and Toxicity; APBS, Adaptive Poisson-Boltzmann Solver; CADD, Computer Aided Drug Discovery; DEGs, Database of essential genes; DAH7PS, 3-deoxy-D-arabino-heptulosonate 7-phosphate; E4P, Erythrose 4-phosphate; FDA, Food and Drug Administration; KEGG, Kyoto Encyclopedia of Genes and Genomes; KAAS, KEGG Automatic Annotation Server; MALT, Mucoid Associated Lymphoid Tissue; MD, Molecular Dynamics; MSA, Multiple Sequence Alignment; NCBI, National Centers for Biotechnology Information; NMR, Nuclear Magnetic Resonance; NPT, Constant Number of particles, Pressure, and Temperature; NVT, Constant Number of particles, Volume, and Temperature; PME, Particle Mesh Ewald; PEP, Phosphoenolpyruvate; PDB, Protein Data Bank; PPI, Proton Pump Inhibitor; Rg, Radius of Gyration; RMSD, Root Mean Square Deviation; RMSF, Root Mean Square Fluctuation; RO5, Lipinski’s Rule of Five; SBVS, Structure Based Virtual Screening; SPC, Simple Point Charge; VDW, Van Der Waals; WHO, World Health Organization.
