# Supplementary material for: Identification of novel drug targets for Helicobacter pylori: structure-based virtual screening of potential inhibitors against DAH7PS protein involved in the shikimate pathway
Source: Front Bioinform. 2024 Oct 18;4:1482338. doi: 10.3389/fbinf.2024.1482338 (PMC11527725; doi:10.3389/fbinf.2024.1482338)
Supplement: Supplementary file 2 [file Table1.docx]

**List of gut microbiota strains:**

1. Actinomyces_odontolyticus_ATCC_17982
2. Akkermansia_muciniphila_ATCC_BAA-835
3. Alistipes_putredinis_DSM_17216
4. Anaerofustis_stercorihominis_DSM_17244
5. Anaerostipes_caccae_DSM_14662
6. Anaerotruncus_colihominis_DSM_17241
7. Pseudoflavonifractor_capillosus_ATCC_29799
8. Bacteroides_cellulosilyticus_DSM_14838
9. Bacteroides_coprocola_DSM_17136
10. Bacteroides_dorei_DSM_17855
11. Bacteroides_eggerthii_DSM_20697
12. Bacteroides_finegoldii_DSM_17565
13. Bacteroides_intestinalis_DSM_17393
14. Bacteroides_ovatus_ATCC_8483
15. Bacteroides_pectinophilus_ATCC_43243
16. Bacteroides_plebeius_DSM_17135
17. Bacteroides_stercoris_ATCC_43183
18. Bacteroides_uniformis_ATCC_8492
19. Bifidobacterium_adolescentis_ATCC_15703
20. Bifidobacterium_adolescentis_L2-32
21. Bifidobacterium_angulatum_DSM_20098
22. Bifidobacterium_bifidum_DSM_20456
23. Bifidobacterium_breve_DSM_20213
24. Bifidobacterium_dentium_ATCC_27678
25. Bifidobacterium_longum_DJO10A
26. Bifidobacterium_longum_NCC2705
27. Bifidobacterium_longum_subsp_infantis_str_ATCC_15697
28. Borrelia_burgdorferi_CA-11.2A
29. Butyrivibrio_crossotus_DSM_2876
30. Catenibacterium_mitsuokai_DSM_15897
31. Clostridium_asparagiforme_DSM_15981
32. Clostridium_bartlettii_DSM_16795
33. Clostridium_bolteae_ATCC_BAA-613
34. Clostridium_hiranonis_DSM_13275
35. Clostridium_leptum_DSM_753
36. Clostridium_methylpentosum_DSM_5476
37. Clostridium_nexile_DSM_1787
38. Clostridium_ramosum_DSM_1402
39. Clostridium_scindens_ATCC_35704
40. Clostridium_sp_L2-50
41. Clostridium_sp_M62/1
42. Clostridium_sp_SS2/1
43. Clostridium_spiroforme_DSM_1552
44. Clostridium_sporogenes_ATCC_15579
45. Clostridium_symbiosum_ATCC_14940
46. Collinsella_aerofaciens_ATCC_25986
47. Collinsella_intestinalis_DSM_13280
48. Collinsella_stercoris_DSM_13279
49. Coprococcus_comes_ATCC_27758
50. Coprococcus_eutactus_ATCC_27759
51. Dorea_formicigenerans_ATCC_27755
52. Dorea_longicatena_DSM_13814
53. Eggerthella_lenta_DSM_2243
54. Enterobacter_cancerogenus_ATCC_35316
55. Eubacterium_dolichum_DSM_3991
56. Eubacterium_hallii_DSM_3353
57. Eubacterium_siraeum_DSM_15702
58. Eubacterium_ventriosum_ATCC_27560
59. Faecalibacterium_prausnitzii_A2-165
60. Faecalibacterium_prausnitzii_M21/2
61. Lactobacillus_salivarius_UCC118
62. Methanobrevibacter_smithii_ATCC_35061
63. Methanobrevibacter_smithii_DSM_11975
64. Methanobrevibacter_smithii_DSM_2374
65. Methanobrevibacter_smithii_DSM_2375
66. Mitsuokella_multacida_DSM_20544
67. Parabacteroides_johnsonii
68. Parabacteroides_merdae_ATCC_43184
69. Parvimonas_micra_ATCC_33270
70. Photorhabdus_luminescens_subsp_laumondii_TTO1
71. Prevotella_copri_DSM_18205
72. Providencia_alcalifaciens_DSM_30120
73. Providencia_rettgeri_DSM_1131
74. Providencia_rustigianii_DSM_4541
75. Roseburia_faecis_M72/1
76. Roseburia_intestinalis_L1-82
77. Ruminococcus_gnavus_ATCC_29149
78. Ruminococcus_lactaris_ATCC_29176
79. Ruminococcus_obeum_ATCC_29174
80. Ruminococcus_torques_ATCC_27756
81. Streptococcus_infantarius_subsp_infantarius_ATCC_BAA-102
82. Subdoligranulum_variabile_DSM_15176
83. Victivallis_vadensis_ATCC_BAA-548
